# AbcApp: incidence of intra-abdominal ABsCesses following laparoscopic vs. open APPendectomy in complicated appendicitis

**DOI:** 10.1007/s00464-022-09670-4

**Published:** 2022-10-06

**Authors:** Bobby Zamaray, M. F. J. de Boer, Z. Popal, A. Rijbroek, F. W. Bloemers, S. J. Oosterling

**Affiliations:** 1 Department of Surgery, Spaarne Gasthuis, Location Haarlem and Hoofddorp, Boerhaavelaan 22, 2035RC Haarlem, The Netherlands; 2 Department of Surgery, Amsterdam University Medical Center, Location AMC and VUmc, Amsterdam, The Netherlands

**Keywords:** Complicated appendicitis, Laparoscopy, Appendectomy, Intra-abdominal abcess

## Abstract

**Background:**

Patients with complicated appendicitis are more at risk for the occurrence of postoperative intra-abdominal abscesses than patients with uncomplicated appendicitis. Studies comparing laparoscopic and open appendectomy showed limitations and contradictory findings on the incidence of intra-abdominal abscesses after appendicitis, as most of these studies analysed both uncomplicated and complicated appendicitis as one group. The aim of the present study is to investigate the incidence of intra-abdominal abscesses after laparoscopic versus open appendectomy for complicated appendicitis.

**Methods:**

A retrospective cohort study was performed over the period January 2009 till May 2020. All patients who had an intra-operative diagnosis of complicated appendicitis (e.g. perforation, necrosis) were included. The outcome measure was the occurrence of intra-abdominal abscesses with a postoperative follow-up of 30 days. Multivariate logistic regression analysis was performed including adjustments for significant confounders.

**Results:**

A total of 900 patients had undergone appendectomy for complicated appendicitis. The majority was operated laparoscopically (78%, *n* = 705). The incidence of postoperative intra-abdominal abscess was 12.3% in both laparoscopic and open appendectomy groups. On univariable analysis, the postoperative rates of intra-abdominal abscesses between laparoscopic and open appendectomy were not significantly different (odds ratio 1.11, 95% CI [0.67–1.84], *p* = 0.681).

**Conclusion:**

The present study provides evidence that, in current daily practice, intra-abdominal abscess formation remains a common postoperative complication for complicated appendicitis. Nonetheless, no significant difference was found with regard to intra-abdominal abscess formation when comparing laparoscopy with open surgery.

Appendectomy is one of the most common performed surgical procedures worldwide. Traditionally, this operation is performed through an open incision as originally described by McBurney in 1894 [1]. With the rise of endoscopic surgery in the early 1990’s, a laparoscopic approach was introduced after the initial description by Semm [2]. Presently, laparoscopic appendectomy (LA) has gained wide acceptance and is becoming the standard approach for appendicitis worldwide. In 2014, approximately, 80% of adult patients presenting with appendicitis in Dutch hospitals were operated using laparoscopy, mainly decided by the preferred surgical expertise of the operating surgeon [3].

Yet, there remains controversy regarding evidence of outcome measures and their clinical importance. Compared to the open approach, LA is associated with a shorter length of stay, fewer postoperative wound infections and less postoperative pain [4]. However, with regard to the incidence of intra-abdominal abscesses (IAA) following surgery, open appendectomy (OA) has shown to be favourable over LA in several studies [4, 5]. Their results, however, are debatable whilst these studies used outdated data from the period that LA was emerging. Furthermore, uncomplicated appendicitis (UA) and complicated appendicitis were analysed as one group, while it is believed that the two have a different pathophysiology [6]. In addition, studies have shown that IAA are mainly seen after appendectomy in complicated appendicitis [3, 7, 8].

With regard to complicated appendicitis, a recent systematic review of Yu MC et al*.* [9] investigated 2 RCTs and 14 retrospective studies comparing LA and OA for complicated appendicitis. Although large numbers of patients are analysed, their studies lack 30-days follow-up data.

Given the limitations and contradictory findings of the above-mentioned studies, the present study aimed to determine differences in incidence of IAA in patients with complicated appendicitis following LA and OA.

## Methods

### Study design

This retrospective cohort study was performed in the Spaarne Gasthuis Haarlem/Hoofddorp, two large general teaching community hospitals in the Netherlands. The database consists of records maintained in our electronic patient system (EPIC). The medical ethics committee of the Spaarne Gasthuis approved the study and determined that informed consent was not necessary, whilst patient identity remained anonymous. All patients (of all ages) undergoing an appendectomy for complicated appendicitis between January 2009 and May 2020 in the Spaarne Gasthuis were selected using a data retrieving programme named CT-Cue [10]. Complicated appendicitis was defined as an appendix with intra-operative characteristics of necrosis/gangrene and/or perforation and/or appendiceal abscess and/or purulent appendicitis. This intra-operative evaluation was conducted by the attending surgeon.

After the first selection by CT-Cue, these patient files were manually screened and selected. Patients treated electively were not included, as well as patients who initially had conservative treatment. Patients who had conversion during surgery were excluded. Also, midline laparotomy, extended resection of the caecum/colon (ileocecal resection or right hemicolectomy) and/or a postoperative pathological diagnosis of carcinoma were excluded. Tourist with a general practitioner in the country of origin were excluded because of lacking re-admittance data.

Patient baseline demographics included the following: sex, age, American Society of Anaesthesiologists (ASA) physical status score, history of diabetes mellitus (type I or II) and history of abdominal surgery (laparoscopic or laparotomy). Intra-operative data included the following: Surgeon or resident, the presence of an intra-operative peri-appendiceal abscess, the presence of purulent appendicitis, the presence of necrotic appendicitis, the presence of peritonitis, the presence of a macroscopic perforation, intra-operative saline irrigation, postoperative days of received intravenous antibiotics and pathological diagnosis (simple inflamed appendicitis, gangrenous appendicitis or perforated appendicitis).

### Definition of outcome measures

The primary outcome measure was the occurrence of IAA within a postoperative follow-up of 30 days. An IAA was defined as a postoperative intra-abdominal fluid collection diagnosed by either ultrasonography or cross-sectional imaging (CT/MRI) in combination with increased biochemical inflammatory values (C-reactive protein (CRP) > 10 mg/l or Leukocyte count > 10^9^/l), which acquired in-hospital treatment (intravenous antibiotics, with or without abscess drainage).

### Data collection

After identification of patients with complicated appendicitis (using CT-Cue), the retrospective data were collected by two researchers (BZ, MB) from the electronic patient database in the Spaarne Gasthuis. To obtain the data needed, operation reports, admission charts and daily status reports were used and anonymously processed in our medical web-based, database. To identify IAA formation, the electronic files of every included patient were checked during the 30-day follow-up. Additionally, data such as medical imaging and procedures were used to identify intra-abdominal abscesses.

### Statistical analysis

SPSS® version 24 (IBM, Armonk, New York, USA) was used to statistically analyse the available data.

Absolute frequencies and percentages were used to analyse qualitative variables. Quantitative variables were analysed as means and standard deviation (STD) because of normal distribution of those variables. Descriptive statistical analysis was used to analyse patient characteristics in the different operation approach groups (LA and OA) using independent t tests for continuous variables and Pearson Chi-Square analysis for categorical variables. To compare the IAA ratio after laparoscopic and open appendectomy, an univariable regression analyses were performed, followed by a multivariable analysis using significant confounders and operation approach. The odds ratio (OR) was calculated using multivariable regression analysis for significant confounders (*P* < 0.05). Finally, a sub-analysis of the period January 2016 to May 2020 was performed to analyse whether recent enhancements in laparoscopy showed less postoperative IAA formation.

## Results

**Fig. 1:**
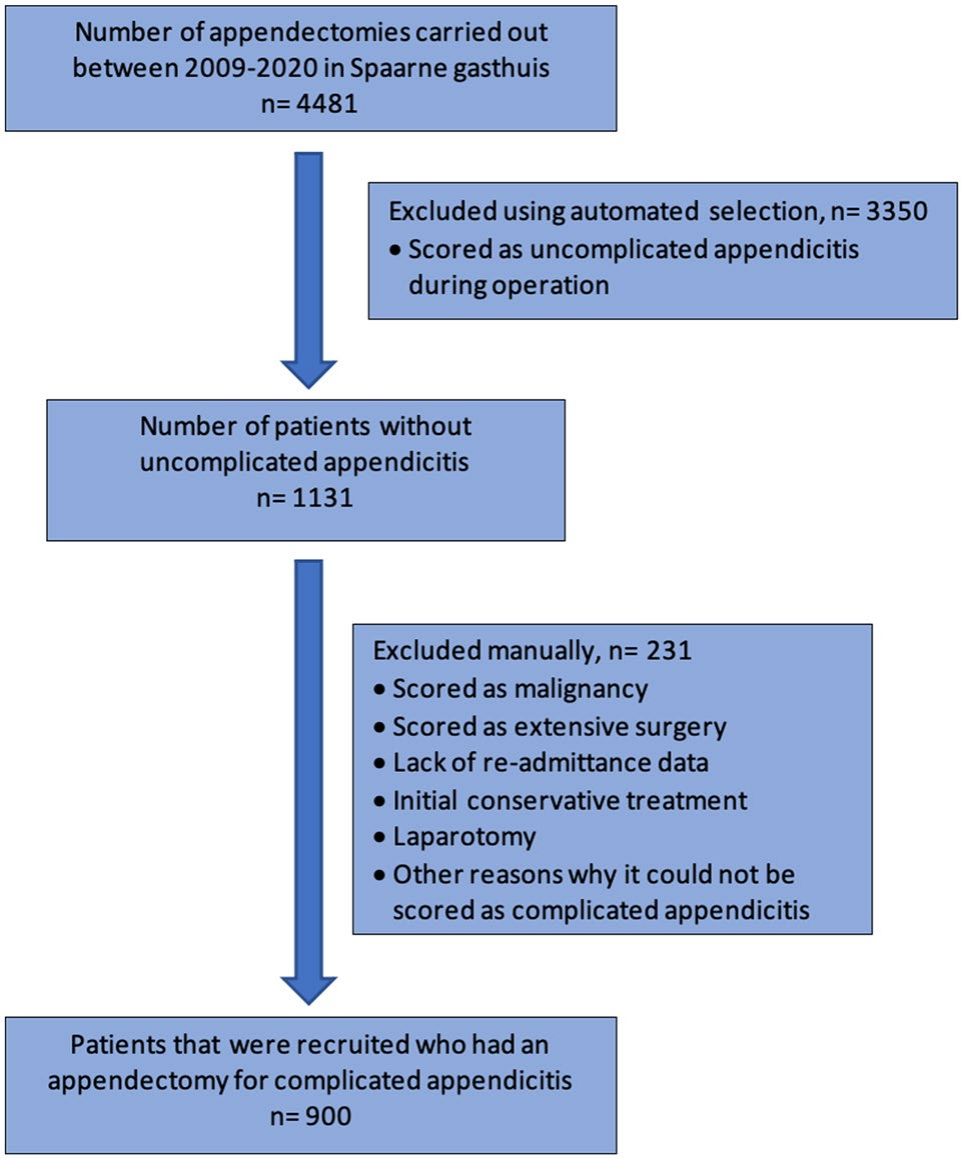
Flow diagram of cohort study population

A total of 4488 patients underwent surgery for acute appendicitis in the Spaarne Gasthuis between January 2009 and May 2020. Of the entire patient group, 1131 patients were suspected to have a complicated appendicitis. A total of 900 patients out of 1131 were selected manually to have met the inclusion criteria and none of the exclusion criteria. Thus, all 900 patients had an appendectomy for acute complicated appendicitis (Fig. 1).

### Patient demographics

The laparoscopy and open group consisted of 705 (78%) and 195 patients (21.7%), respectively (Table 1). The overall mean age was 38.4 years (range 2–92, SD 22.5), with 483 male patients (53.7%). In the open appendectomy group, male patients were relatively more represented than female patients. The percentage of patients having a history of diabetes mellitus was significantly different, with 12 patients (6.2%) in the open group and 20 patients (2.8%) in the laparoscopic group. There was only one patient with an ASA score of 4 in the laparoscopic group, but there were no significant differences in ASA scores, nor in age, between both study groups. Additionally, there was no statistically significant difference in type of surgeon operating (consultant, consultant with resident or resident alone) between the two groups (Table 2, *P* = 0.078).

**Table 1:** Baseline demographics and preoperative data for patients undergoing appendectomy for complicated appendicitis

Parameter	Total n (%) *n* = 900	Laparoscopic appendectomy n (%) *n* = 705 (78.3%)	Open appendectomy *n *(%) *n* = 195 (21.7%)	*P-*value
Sex ratio, Male: Female	483: 417 (53.7%/46.3%)	366: 339 (51.9%/48.1%)	117: 78 (60.0%/40.0%)	0.045
Age, Mean (SD)	38.4 (22.5)	38.8 (21.7)	37.1 (25.2)	0.353
ASA classification				
≥ III	36 (4%)	26 (3.7%)	10 (5.1%)	0.654
II	256 (28.4%)	198 (28.1%)	58 (29.7%)	
I	608 (67.6%)	481 (68.2%)	127 (65.1%)	
Diabetes mellitus	32 (3.6%)	20 (2.8%)	12 (6.2%)	0.027
History of abdominal surgery	107 (11.9%)	80 (11.3%)	27 (13.8%)	0.340

*ASA* American Society of anaesthesiology, *SD* standard deviation

**Table 2:** Operative data for patients undergoing appendectomy for complicated appendicitis

Parameter	Laparoscopic appendectomy (*n* = 705 (78.3%))	Open appendectomy (*n* = 195 (21.7%))	*P-*value
Surgeon			
Consultant alone	174 (24.7%)	56 (28.7%)	0.078
Consultant or resident under supervision	470 (66.7%)	131 (67.1%)	
Resident without supervising consultant	61 (8.7%)	8 (4.1%)	
Intra-operative diagnosis			
Peri-appendiceal abscess	81 (11.5%)	22 (11.3%)	0.936
Purulent appendicitis	505 (71.6%)	124 (63.5%)	0.030
Gangrenous appendicitis	364 (51.6%)	81 (41.5%)	0.013
Peritonitis	72 (10.2%)	5 (2.6%)	0.001
Macroscopic perforation	374 (53.0%)	123 (63.1%)	0.013
Intra-operative flushing	473 (67.1%)	125 (64.1%)	0.434
Postoperative IV antibiotics, Days (SD)	3.25 (1.71)	3.58 (1.53)	0.013
Pathology diagnosis			
Simple inflamed appendix	411 (59.6%)	104 (55.3%)	0.389
Gangrenous appendix	141 (20.4%)	51 (27.1%)	
Perforated appendix	10 (1.4%)	3 (1.6%)	
Simple inflamed + gangrenous	23 (3.3%)	5 (2.7%)	
Simple inflamed + perforated	93 (13.5%)	24 (12.8%)	
Simple inflamed + gangrenous + perforated	12 (1.7%)	1 (0.5%)	

### Outcome measures

Intra-operative findings showed more purulent appendicitis (71.8% vs. 63.1%, *P* = 0.030), more gangrenous appendicitis (51.6% vs. 41.5%, *P* = 0.013) and more abdominal peritonitis (10.2% vs. 2.6%, *P* = 0.001) in the laparoscopy group. In the open group, more macroscopic perforations were seen (63.1% vs. 53.0%, *P* = 0.013). Postoperative histologic reports did not differ between the open and laparoscopy group. The mean duration of intravenous antibiotics treatment was 3.25 days (STD = 1.71) in the laparoscopic group and 3.58 days (STD = 1.56) in the open group (*P* = 0.013), meaning a significant difference between both groups.

The rate of intra-abdominal abscess formation did not significantly differ between the two groups (Table 3) with an incidence of 12.3% in both groups. A sub-analysis of the period January 2016 to May 2020 also demonstrated a non-significant difference between laparoscopic appendectomy and open appendectomy (respectively 13.4% vs 11.6%, *P* = 0.74). The significant difference “Postoperative IV antibiotics” (*P* = 0.013) was classified as a potential confounder, for the occurrence of IAA, and used in the multivariable model with operation “Approach” (Table 3) to calculate the OR: 1.08 (95% CI [0.66–1.77], *P* = 0.75). The variable “Approach” remained a non-significant predictor for IAA.

**Table 3:** Univariable and multivariable analysis of risk factors for intra-abdominal abscess

Variable	Univariable analysis		Multivariable analysis	
	OR [95% CI]	P	OR (95% CI)	P
Sex				
Female	Reference 0.98 [0.66–1.46]	0.91		
Male				
Diabetes mellitus				
No	Reference 1.02[0.35–2.95]	0.98		
Yes				
Approach				
Open	Reference 1.00[0.62–1.63]	0.99	Reference 1.08 [0.66–1.77]	0.75
Laparoscopic				
Postoperative IV antibiotics, Days				
Mean	1.33 [1.18–1.50]	< 0.001	1.33 [1.18–1.50]	< 0.001

Furthermore, 46% of patients who developed intra-abdominal abscesses were diagnosed during primary admission. The remaining 54% developed their abscess after initial discharge and were re-admitted to the hospital in the 30 days follow-up period (Table 4).

**Table 4:** Diagnosis of IAA and re-admittance rate in patients with complicated appendicitis

	Intra-abdominal abscess (*n* = 111)
Diagnosis of IAA AFTER primary admission, and re-admission needed	60 (54%)
Diagnosis of IAA DURING primary admission	51 (46%)

## Discussion

In the present study, there was a 12.3% incidence of postoperative IAA in patients with complicated appendicitis. Patients with an IAA received more intravenous antibiotics, in days. The incidence of IAA did not significantly differ following LA and OA. The laparoscopic operation technique was used in 78% of patients. Purulent appendicitis, gangrenous appendicitis and abdominal peritonitis were seen more frequent in the laparoscopy group, whereas macroscopic perforations were seen more often during OA.

An intra-abdominal abscess is a postoperative complication with high morbidity. It is associated with prolonged length of hospital admission, re-admissions, re-intervention and high medical costs [8]. Complicated appendicitis is strongly associated with IAA [8]. Considering 16.000 appendectomies that are performed yearly in the Netherlands [11], of which 20–30% is complicated [3, 12, 13], 3.200–4.800 appendectomies are carried out for complicated appendicitis. In our study, 12.3% of the group developed an IAA, thus an estimated 400–500 patients a year develop this complication in the Netherlands. Despite the large number of patients developing IAA after appendectomy for complicated appendicitis, no randomized clinical trials with large patient sample sizes have been conducted to compare an open appendectomy with a laparoscopic procedure in complicated appendicitis.

The most recent Cochrane review by Jaschinski et al. [14] showed intra-abdominal abscess rates of 1.2% (40/3333 patients) in open appendectomy compared to 1.9% (66 out of 3334 patients) for laparoscopic appendectomy [11]. In this systematic review, both uncomplicated and complicated appendicitis were analysed as one group. The incidence of IAA in our study, analysing only complicated appendicitis, is about tenfold higher. Comparing the incidence rates in this study and the systematic review illustrates the strong association of IAA with complicated appendicitis.

A recent study by van Rossem et al. analysed patients with complicated appendicitis [3]. They concluded that there was no significant difference between LA (10.2%) and OA (6.0%) in complicated appendicitis [3]. However, their results demonstrated an OR of 1.69 with a wide CI (95% CI [0.69–4.14], *p* = 0.254) [3]. This made us question whether the study was adequately powered to draw a conclusion about abscess formation after complicated appendicitis. The results of the present study now support their findings. As mentioned before, the IAA rate in the present study was 12.3% in both LA and OA with an OR: 1.08 (95% CI [0.66–1.77], *P* = 0.75) after correction for duration of “Post-operative IV antibiotics”. Therefore, our data demonstrated no statistically significant difference in IAA formation between LA and OA in complicated appendicitis.

The systematic review of Yu et al. [9] comparing laparoscopic versus open appendectomy in complicated appendicitis included 15 (of 16) studies that showed no significant difference in postoperative IAA formations [9], in accordance with our study. Many of the included trials, however, had small sample sizes. The two studies with meaningful sample sizes were Tuggle et al. and Masoomi et al. Tuggle et al. found a significant difference (*P* < 0.001) in incidence of IAA within the laparoscopic and open appendectomy groups. With IAA rates of 3.69% (26 out of 730 patients) for the OA group and 6.74% (130 out of 2060 patients) in the LA group, they indicated an increased risk of IAA after laparoscopy [13]. In contrast, Masoomi et al. (138.000 inclusions) reported no significant difference in incidence of IAA in complicated appendicitis between the laparoscopy and open group, with IAA rates of 1,7% in the LA group and 3,6% in the OA group [12]. Thorough analysis of their data showed that they used the National inpatient sample (NIS) data, which in our opinion is sensitive to inaccuracy in data collection. Moreover, the cause of the low incidence of IAA in their studies might be caused by the lack of re-admission data. Our present results illustrate the importance of re-admission data for the incidence and clinical impact of IAA, with approximately half of the IAA being diagnosed after primary discharge.

It is believed that open surgery and conversion are more often performed in the complex cases and by more skilled surgeons, this could cause selection bias. For this reason, we excluded conversion during surgery, midline laparotomy and extended resection of the caecum/colon. Thus, the open appendectomy performed was based on a simple incision in the right lower quadrant of the abdomen. Therefore, we believe that patients in both the laparoscopy and open group are operated by surgeons with comparable skill for these procedures.

The difference in intra-operative findings (e.g. pus, peritonitis, macroscopic perforation) between LA and OA can be explained by the used operation technique. In laparoscopy, the surgeon might have better view of the abdominal details. On the other hand, when performing OA, the surgeon might have more detailed vision of (extracted) appendix for it is not directly sealed in an endobag.

The strength of the present study includes the focus on a more specific study population (namely patients with complex appendicitis), combined with the relatively large patient sample size and in particular the presence of re-admittance data. Furthermore, these major tertiary teaching hospitals of the Netherlands have a heterogeneous patient population, which makes it possible to consider the results as broadly representative for the average patient population (with complex appendicitis) in our country.

We are also aware of the limitations of the present study, which are mainly related to the retrospective nature of the study. Also, the interobserver variation between surgeons, when classifying the appendicitis as complicated or uncomplicated, is a limitation in the present study.

Furthermore, when evaluating the appendix, pathologists classify the microscopic findings they visualize. However, abdominal pus, peritonitis or other abdominal pathologies will not be mentioned in their report, as is the case in the surgery report. We therefore believe that only reviewing the pathology report is not sufficient when evaluating the severity of the disease, but in both cases misclassification bias cannot be eliminated.

## Conclusion

This study provides evidence that in complicated appendicitis, laparoscopic and open appendectomy are equivalent in terms of incidence of postoperative intra-abdominal abscesses (IAA). The complication IAA is and remains a common and associated complication after appendectomy for complicated appendicitis.
